# Plasma and parasternal subcutaneous tissue pharmacokinetics of ceftaroline fosamil in cardiac surgery with cardiopulmonary bypass

**DOI:** 10.3389/fphar.2026.1798506

**Published:** 2026-03-31

**Authors:** Maximilian Edlinger-Stanger, Felix Bergmann, Walter Jaeger, Michaela Boehmdorfer, Markus Zeitlinger, Doris Hutschala-Kinstner

**Affiliations:** 1 Department of Anesthesia, Intensive Care Medicine and Pain Medicine, Division of Cardiac Thoracic Vascular Anesthesia and Intensive Care Medicine, Medical University of Vienna, Vienna, Austria; 2 Department of Clinical Pharmacology, Medical University of Vienna, Vienna, Austria; 3 Department of Pharmaceutical Chemistry, University of Vienna, Vienna, Austria

**Keywords:** cardiac surgery, cardiopulmonary bypass, ceftaroline, microdialysis, MRSA, pharmacokinetics, subcutaneous

## Abstract

Surgical site infections remain significant complications after cardiac surgery and perioperative antibiotic exposure may be altered by cardiopulmonary bypass and patient characteristics. Ceftaroline fosamil is active against methicillin resistant *Staphylococcus aureus* (MRSA) and may be a potential alternative for patients with risk factors for MRSA. However, parasternal subcutaneous tissue exposure during cardiac surgery has not been studied. This prospective, randomized pharmacokinetic study investigated plasma and parasternal subcutaneous concentrations in patients undergoing cardiac surgery with cardiopulmonary bypass. Fourteen patients were randomized to receive a total dose of 1,800 mg of ceftaroline fosamil administered either intermittently or by continuous infusion following a loading dose. Plasma and subcutaneous tissue concentrations were measured for 24 h using arterial blood sampling and *in vivo* microdialysis. Pharmacokinetic parameters and pharmacodynamic target attainment were assessed for both dosing regimens. In plasma, free drug concentrations remained above the MIC of 1 mg/L for nearly the entire dosing interval in both groups (mean *f*T_>MIC_ ∼99–100%). Subcutaneous parasternal tissue concentrations were lower in both groups and showed marked interindividual variability. At an MIC of 1 mg/L, mean tissue *f*T_>MIC_ ranged from 93% to 99% with intermittent and 75%–86% during continuous administration. These findings demonstrate that ceftaroline penetration into subcutaneous parasternal tissue is highly variable in cardiac surgery patients and may limit tissue exposure under perioperative conditions despite favorable plasma concentrations. While continuous infusion improves plasma exposure, this does not necessarily translate into superior tissue exposure. Therefore, measurement of tissue concentrations is important when evaluating antibiotic dosing strategies in the perioperative setting.

## 1 Introduction

Surgical site infections remain significant complications of cardiac surgery and are associated with substantial morbidity and mortality, particularly in cases of deep-seated infections (Lepelletier et al., 2013; Goh, 2017; Sears et al., 2016). Methicillin-resistant *Staphylococcus aureus* (MRSA) is responsible for up to 5% of infections after cardiac surgery (Fowler et al., 2003; Zangrillo et al., 2006). Patients at risk for MRSA infections frequently receive glycopeptides such as vancomycin for antibiotic prophylaxis (Abu-Omar et al., 2017; Sousa-Uva et al., 2017; Calderwood et al., 2023). However, vancomycin may be less effective against methicillin-susceptible *Staphylococcus aureus* than β-lactam agents, such as cefazolin, and is associated with postoperative acute kidney injury (Hutschala et al., 2009; Branch-Elliman et al., 2017) and suboptimal tissue penetration (Martin et al., 1994; Skhirtladze et al., 2006; Kollef, 2007; Payne et al., 2011).

Ceftaroline fosamil, a fifth-generation cephalosporin with activity against MRSA, is a prodrug that is rapidly hydrolyzed to ceftaroline. Ceftaroline undergoes minimal metabolism, is primarily renally excreted and exhibits low, concentration-dependent protein binding (14%–28%) (Jorgenson et al., 2011; Kiang et al., 2015). Similar to other beta-lactam antibiotics, its antibacterial activity correlates with the percentage of the dosing interval during which free drug concentrations exceed the minimum inhibitory concentration (% *f*T_>MIC_) (Andes and Craig, 2006; Lodise et al., 2006). Ceftaroline fosamil is approved for administration as an intermittent infusion. Given the time-dependent pharmacodynamic profile, continuous infusion has been explored as a strategy to optimize % *f*T_>MIC_ (Edlinger-Stanger et al., 2021; Chauzy et al., 2022).

In phase III trials (CANVAS 1, CANVAS 2, COVERS), ceftaroline demonstrated similar cure rates as vancomycin plus aztreonam in complicated skin and soft tissue infections (Corey et al., 2010; Wilcox et al., 2010; Dryden et al., 2016). Likewise, the CAPTURE registry data show high clinical cure rates across various patient populations (Santos et al., 2013; Evans et al., 2014; Lipsky et al., 2015; Maggiore et al., 2015; Welte et al., 2019). In a recent network meta-analysis, ceftaroline showed the second-best effectiveness and microbial killing rate for complicated skin and soft tissue infections after linezolid (Ju et al., 2024). These findings support the efficacy of ceftaroline for MRSA infections, but data on the efficacy for surgical prophylaxis remain limited, particularly with respect to perioperative tissue exposure.

Ceftaroline achieved adequate plasma and tissue (subcutaneous, muscular) concentrations in healthy volunteers with currently approved dosing regimens (Riccobene et al., 2013; Justo et al., 2015; Matzneller et al., 2016). However, tissue penetration may be substantially reduced during cardiac surgery due to altered microcirculation and interstitial fluid shifts during and after cardiopulmonary bypass (CPB), which may limit tissue exposure of ceftaroline in these patients. In a previous study, cefazolin penetration into subcutaneous parasternal tissue was suboptimal and significantly affected by internal mammary artery harvesting (Andreas et al., 2013). Ceftaroline showed adequate penetration into lung tissue during cardiac surgery (Edlinger-Stanger et al., 2021), however, data on subcutaneous parasternal tissue penetration are lacking.

We therefore investigated plasma and parasternal subcutaneous tissue pharmacokinetics of ceftaroline using *in vivo* microdialysis in patients undergoing elective cardiac surgery. To assess whether differences in plasma exposure translate into differences in tissue exposure, patients were randomized to intermittent infusion or continuous administration. This study may provide the pharmacokinetic basis for evaluating the suitability of ceftaroline for perioperative use in cardiac surgery patients.

## 2 Materials and methods

### 2.1 Ethics

This prospective pharmacokinetic study was conducted at the Department of Cardiothoracic and Vascular Anesthesia and Intensive Care Medicine at the Medical University of Vienna, Vienna, Austria, in accordance with current International Conference on Harmonization-Good Clinical Practice (ICH-GCP) guidelines, the Declaration of Helsinki and national and institutional standards. The study was registered under EudraCT number 2017-002508-29 (https://www.clinicaltrialsregister.eu/ctr-search/trial/2017-002508-29/AT), approved by the Ethics Committee of the Medical University of Vienna (EC number 1752/2017), and authorized by the Austrian Agency for Health and Food Safety. Signed informed consent to study participation was obtained from all patients before inclusion.

### 2.2 Patients

Prior to inclusion, written informed consent was obtained preoperatively after detailed instructions about the conduction of the study. Inclusion criteria were: planned coronary bypass grafting (CABG) with left internal mammary artery bypass, planned use of cardiopulmonary bypass, age between 18 and 90 years, and left ventricular ejection fraction >40%. Exclusion criteria were known allergy to penicillin/cephalosporins or ceftaroline, preoperative antibiotic therapy, preoperative signs of infection, emergency procedure, re-operation, planned bilateral internal mammary artery harvesting, preoperative renal failure, chronic severe renal insufficiency including hemodialysis, chronic severe liver disease, BMI >35 and long-standing diabetes mellitus >7 years or insulin-dependent diabetes mellitus.

### 2.3 Materials and substances

Ceftaroline fosamil (Zinforo®) was purchased from Pfizer (Pfizer Corporation Austria GmbH), ceftaroline hydrochloride was purchased from AstraZeneca (AstraZeneca Österreich, Vienna, Austria), Physiological 0.9% saline solution was purchased from Medica Medicare, Kufstein, Austria. Microdialysis catheters were purchased from M Dialysis, Stockholm, Sweden.

### 2.4 Anesthesia and cardiopulmonary bypass

After the establishment of routine monitoring (electrocardiogram, pulse oximetry, arterial blood pressure monitoring), anesthesia was induced with midazolam 0.05–0.1 mg/kg, fentanyl 2–3 μg/kg, propofol 1–3 mg/kg and cis-atracurium 0.2 mg/kg 5–10 mL/kg of Ringer’s lactate solution was administered during induction of anesthesia. Surgery was conducted via a full median sternotomy on CPB. Full heparinization for CPB was achieved with 400 IU/kg heparin sodium, and activated clotting time was kept above 400s (Hemochrom 400; International Technidyne, Edison, New Jersey) during CPB. The CPB priming solution contained Ringer’s lactate solution (1,000 mL), hydroxyethyl starch 6% (500 mL), and 10.000, IE heparin sodium. Body core temperature was maintained at 36 °C. After successful weaning from CPB, heparin was antagonized with protamine.

### 2.5 Administration of ceftaroline fosamil

Patients were randomized using a sealed envelope system to receive ceftaroline fosamil either by intermittent administration or by continuous infusion following a loading dose. Patients in the intermittent group received 600 mg every 8 h over 120 min. In the continuous group, a loading dose of 600 mg was administered over 120 min, followed by a continuous infusion of ceftaroline fosamil (1,200 mg over 22 h). All patients received a total intravenous dose of 1,800 mg of ceftaroline fosamil. The first dose was administered following the 30 min run-in period after microdialysis probe insertion.

Adverse events were assessed by clinical monitoring during the perioperative and postoperative period and documented by the treating clinical team according to routine clinical practice.

### 2.6 *In vivo* microdialysis

Microdialysis allows the measurement of free, unbound drug concentrations in various tissues. This method has been described previously in detail (Chaurasia et al., 2007; Riccobene et al., 2013). During the sampling period, microdialysis catheters are perfused with perfusion solution at a low rate. The perfusion solution reaches the semipermeable microdialysis membrane via the inlet tube. Analytes diffuse across the semipermeable microdialysis membrane depending on the concentration gradient. The dialysate is collected at the outlet tube and analyzed subsequently.

63 GI Microdialysis Catheters (M Dialysis AB, Stockholm, Sweden) with a membrane length of 10 mm and a molecular mass cutoff of 20.000 Daltons, a polyurethane tubing and pulyarylethersulphone membrane were used.

During induction of anesthesia, microdialysis probes were inserted subcutaneously left and right to the sternal midline (approximately 5 cm parasternal). Thereafter, probes were perfused with 0.9% saline solution at a flow rate of 2 μL/min using a microinfusion pump.

### 2.7 Sampling intervals

**FIGURE 1:**
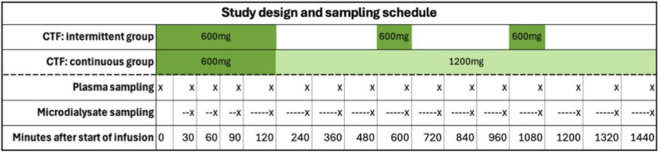
Study design and sampling schedule for patients in the intermittent and continuous group. Timing of CPB varied between patients and is therefore not shown. Plasma sampling time points are indicated by crosses (x). Microdialysis samples, which represent analyte collection over predefined time intervals, are illustrated by dashed lines ending in a cross (---x). CTF, ceftaroline fosamil.

Plasma concentrations of ceftaroline were measured at baseline, after 30 min, 60 min, 90 min, and 120 min and every 2 h thereafter. Microdialysis samples were collected in 30 min intervals for the first 2 h and every 2 h thereafter. The total sampling duration was 24 h. Figure 1 shows dosing regimens and sampling time points for both groups.

### 2.8 Probe calibration

Individual microdialysis probes were calibrated using the retrodialysis method (Chaurasia et al., 2007). The perfusion solution for retrodialysis contained ceftaroline hydrochloride at a concentration of 30 μg/mL. After a 30 min run-in period, the mean recovery of two consecutive 30 min retrodialysis samples was calculated.

Relative recovery (RR) was calculated as follows:

$$\text{RR }\left(\%\right)=100-100x\left({\left[\text{analyte}\right]}_{\text{dialysate}}/{\left[\text{analyte}\right]}_{\text{perfusate}}\right)$$

[Analyte]_dialysate_ is the concentration in the collected retrodialysis sample, [analyte]_perfusate_ is the concentration used in the perfusion solution. “True” extracellular fluid analyte concentrations are calculated as follows:

$${\left[\text{analyte}\right]}_{\text{extracellular}\;\text{fluid}}=100\;x\;\left(\left[\text{microdialysis sample}\right]/\text{RR}\%\right)$$

### 2.9 Sample handling and analysis

4 mL of arterial blood were collected at each time point. Blood samples were immediately placed on ice and centrifuged for 15 min at 3,500 rpm (4 °C) within 15 min. Thereafter, 2 mL plasma were transferred into cryovials, immediately placed on ice and stored at approximately −80 °C within 15 min from sampling until analysis.

The concentration of ceftaroline in plasma and microdialysis was determined by high-performance liquid chromatography (HPLC) using a Dionex UltiMate 3,000 system (Thermo Fisher Scientific, Inc., Waltham, MA) with UV detection at 243 nm. Frozen plasma samples were thawed at room temperature. After the addition of 200 µL ice-cold acetonitrile to 100 µL plasma, the samples were centrifuged (13000 g for 5 min at 4 °C) and 80 µL of the supernatant were injected onto a Hypersil BDS C18 column (5 μm, 250 × 4.6 mm I.D., Thermo Fisher Scientific, Waltham, MA) preceded by a Hypersil BDS-C18 guard column (5 μm, 10 × 4.6 mm I.D.) at a flow rate of 1 mL/min. Microdialysate samples (50 µL) were injected onto the column without any previous precipitation procedure. The column oven was at 35 °C. The mobile phase consisted of a continuous gradient mixed from ammonium acetate buffer (10 mM, pH = 5.0) (mobile phase A) and acetonitrile (mobile phase B). Mobile phase B linearly increased from 5% (0 min) to 25% at 15 min, further increased to 80% at 16 min and was kept constant at 80% until 20 min. The percentage of acetonitrile was then decreased to 25% within 1 min to equilibrate the column for 9 min before injecting of the next sample. Quantification of ceftaroline was based on external calibration curves of spiked drug-free human plasma and microdialysate (pooled patient samples at time 0) with ceftaroline at concentrations ranging from 0.01 to 30 μg/mL (average correlation coefficients >0.998). The limit of quantification of ceftaroline in plasma and microdialysate was 0.02 μg/mL and 0.01 μg/mL respectively (coefficients of accuracy and precision were <9%).

### 2.10 Determination of ceftaroline protein binding

Aliquots (500 µL) of plasma of each patient (collected 1 h after ceftaroline application) were transferred to Centrisart I Ultrafiltration Devices (Sartorius Stedim Biotech S.A., Aubagne, France) and centrifuged at 13,000 *g* for 30 min at room temperature. Subsequently, the recovered ultrafiltrate was analyzed by HPLC as described above to determine the concentration of free (unbound) drug. To determine the total drug concentration (bound and unbound), 200 µL of ice-cold acetonitrile was added to 100 µL aliquots of the same plasma samples from each patient. The samples were then centrifuged, and the supernatant was analyzed by HPLC, as described above. Protein binding of ceftaroline was then calculated according to the following equation:

$$\%\text{ protein binding}=100\;x\;\left(\text{total}-\text{unbound}\right)/\left(\text{total}\right)$$

### 2.11 PK/PD analysis and statistical analysis

Pharmacokinetic parameters were calculated using non-compartmental analysis (NCA) with the R package “NonCompart” (version 0.7.0). Total drug concentrations were adjusted for protein binding, as quantified by ultrafiltration, to derive unbound (free) plasma concentrations. In the intermittent group, AUC_ss_ refers to measurements under assumed steady state conditions (i.e., from the third dose onwards). Subsequently, the AUC_ss__0–8_ was extrapolated to the AUC_ss__0–24_ by multiplying by a factor of 3. In the continuous infusion group, the average concentrations under assumed steady-state conditions (C_ss average_), estimated from samples obtained between 16 and 24 h, were multiplied by 8 and 24 to derive the AUC_ss 0–8_ and AUC_ss 0–24_, respectively.

For beta-lactams, the percentage of the dosing interval during which free drug concentrations exceed the minimum inhibitory concentration (% *f*T_>MIC_) correlates with efficacy (Andes and Craig, 2006; Lodise et al., 2006). According to clinical and experimental data, a target range of 50%–100% *f*T_>MIC_ is considered clinically relevant (MacGowan et al., 2013; Das et al., 2019; Berry and Kuti, 2022). Therefore, 50% and 100% *f*T_>MIC_ were chosen for our analysis to cover both ends of this range.

% *f*T_>MIC_ was calculated for plasma and subcutaneous tissue concentrations under assumed steady-state conditions. An MIC value of 1 mg/L was selected based on MIC distribution data reported by the European Committee on Antimicrobial Susceptibility Testing (EUCAST) and represents the epidemiological cutoff and current susceptibility breakpoint for MRSA (Das et al., 2019; The European Committee on Antimicrobial Susceptibility Testing, 2025). Additionally, the proportion of participants reaching the PK/PD target of 50% and 100% *f*T_>MIC_ was determined separately for plasma and subcutaneous tissue concentrations and for both dosing regimens at MICs of 0.125–16 mg/L. The reported proportions are descriptive and based on observed concentrations.

Linear interpolation was applied between adjacent sampling timepoints to estimate % *f*T_>MIC_. Missing concentration values between observed timepoints were imputed using linear interpolation. Subjects with missing concentrations at the beginning or end of the evaluated interval were excluded from the analysis, as extrapolation beyond observed data was not performed. Participants missing concentration data for more than one time point within the evaluated interval were excluded from the analysis of the proportion of patients reaching a prespecified PK/PD target.

Statistical analysis and visualizations were performed using R (version 4.1.2, 2021, Vienna, Austria) and GraphPad Prism (version 10.4.0), respectively.

## 3 Results

### 3.1 Study population

We included a total of fourteen patients. Seven patients were randomized to each treatment group. Seven patients underwent isolated CABG, three patients had concomitant valve surgery, and one patient had concomitant replacement of the aortic valve and ascending aorta. All operations were performed with CPB. All fourteen patients completed the study. Table 1 shows the summary of patient demographics and relevant intra- and postoperative data.

**TABLE 1:** Demographic and intraoperative data.

Characteristic	Intermittent group (n = 7)	Continuous group (n = 7)
Age (years)	66 ± 12	67 ± 13
Sex (male)	6 (86%)	7 (100%)
Height (cm)	173 ± 6	176 ± 9
Body weight (kg)	84 ± 14	88 ± 15
BMI (kg/m^2^)	28 ± 4	28 ± 4
CPB time (min)	138 ± 76	133 ± 55
ACC time (min)	79 ± 38	80 ± 39
Serum creatinine (mg/dL)	1.0 ± 0.2	1.1 ± 0.2

Data are presented as mean ± SD. ACC, aortic cross clamp; BMI, body mass index; CPB, cardiopulmonary bypass.

### 3.2 Pharmacokinetic analysis

**FIGURE 2:**
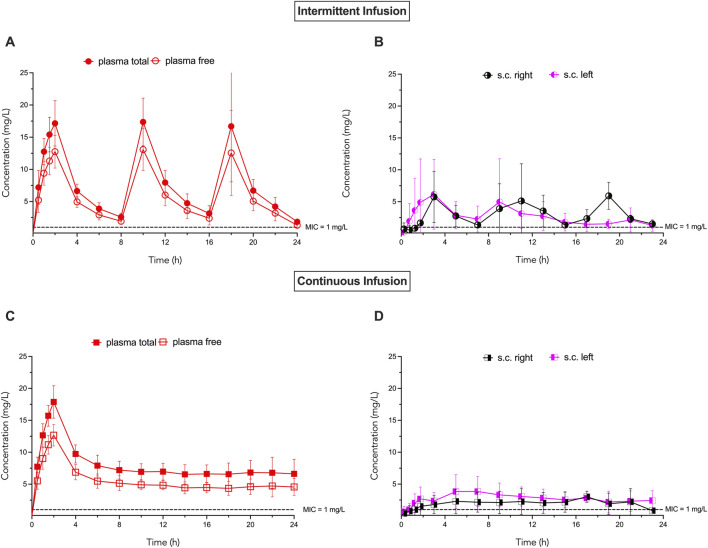
Concentration-time profiles for ceftaroline in plasma and parasternal subcutaneous tissue in the intermittent and continuous group. **(A)** Total and free plasma ceftaroline concentrations in the intermittent group. **(B)** Free ceftaroline concentrations in left and right parasternal subcutaneous tissues in the intermittent group. **(C)** Total and free plasma ceftaroline concentrations in the continuous group. **(D)** Free ceftaroline concentrations in left and right parasternal subcutaneous tissues in the continuous group. Data points represent mean ± standard deviation. MIC = minimum inhibitory concentration. An MIC of 1 mg/L was chosen based on the ECOFF value for MRSA according to EUCAST.

Mean concentration-time profiles of total and free ceftaroline in plasma and free ceftaroline in left and right parasternal subcutaneous tissues for the intermittent and continuous group are shown in Figure 2. Data are reported as mean ± SD. PK data are summarized in Table 2.

**TABLE 2:** Pharmacokinetic parameters for ceftaroline in plasma and parasternal subcutaneous tissues for intermittent infusion and continuous infusion^a^.

Intermittent group			Continuous group		
Plasma	Total	Free	Plasma	Total	Free
C_max ss_ (mg/L)	15.7 ± 8.01	11.9 ± 6.2	C_average ss_ (mg/L)	6.9 ± 2.0	4.5 ± 1.1
AUC_ss 0–8_ (mg*h/L)	52.5 ± 19.8	39.5 ± 15.6	AUC_ss 0–8_ (mg*h/L)	46.0 ± 19.9	31.3 ± 13.6
AUC_ss 0–24_ (mg*h/L)	157 ± 59.5	118 ± 46.8	AUC_ss 0–24_ (mg*h/L)	138 ± 59.7	93.8 ± 40.8
Protein binding (%)	25.2 ± 6.7	NA	Protein binding (%)	28.8 ± 6.8	NA
T > MIC_ss_ (%)^b^	100 ± 0	99.6 ± 0.7	T > MIC_ss_ (%)^b^	100 ± 0	100 ± 0
t_1/2_ (h)	2.4 ± 0.2	NA	​	​	​
CL (L/h)	13.1 ± 5.4	NA	​	​	​
V_d_ (L)	30.1 ± 21.0	NA	​	​	​

Parasternal s.c.	Left	Right	Parasternal s.c.	Left	Right
C_max ss_ (mg/L)	2.6 ± 1.5	5.3 ± 2.3	C_average ss_ (mg/L)	2.7 ± 1.4	2.5 ± 1.6
AUC_ss 0–8_ (mg*h/L)	12.7 ± 6.7	21.8 ± 4.4	AUC_ss 0–8_ (mg*h/L)	16.2 ± 11.6	12.1 ± 9.2
AUC_ss 0–24_ (mg*h/L)	38.2 ± 20	65.5 ± 13.1	AUC_ss 0–24_ (mg*h/L)	48.5 ± 34.8	36.4 ± 27.5
T > MIC_ss_ (%)^b^	93.0 ± 9.6	98.7 ± 2.2	T > MIC_ss_ (%)^b^	75 ± 50	86.0 ± 26.6

^a^ Data are presented as the mean ± standard deviation. NA, not available; AUC_SS, 0–8_, steady-state area under the concentration-time curve from 0 to 8h; AUCss, 0-24, steady-state area under the concentration-time curve from 0 to 24 h; t1/2, half-life; CL, clearance; V_d_, volume of distribution; T > MIC_ss_, proportion of time for which drug concentration remained above the MIC, during the dosing interval; C_max ss_, maximum concentration at steady state; C_max_, average maximum concentration at steady state; s. c., subcutaneous.

^b^ Assuming an MIC, of 1 mg/L for MRSA.

Ceftaroline fosamil was well tolerated by all study subjects without adverse events.

### 3.3 Plasma pharmacokinetics

In the intermittent group, ceftaroline bound and unbound peak plasma concentrations during steady state (C_max SS_) were 15.7 ± 8 mg/L and 11.9 ± 6.2, respectively. The steady state area under the concentration-time curve from 0 to 24 h (AUC_SS 0–24_) was 157 ± 59.5 (mg x h)/L for total plasma concentrations and 118 ± 46.8 (mg x h)/L for unbound plasma concentrations. The apparent volume of distribution during steady state was 30.1 ± 21.0 L. In the continuous group, average steady state concentrations were 6.9 ± 2.0 mg/L for total ceftaroline and 4.5 ± 1.1 mg/L for unbound ceftaroline. The AUC_SS 0–24_ was 138 ± 59.7 (mg x h)/L for total concentrations and 93.8 ± 40.8 (mg x h)/L for unbound concentrations. Plasma protein binding was 25.2% ± 6.7% in the intermittent group and 28.8% ± 6.8% in the continuous group. The difference in AUC_SS 0–8_, AUC_SS 0–24_, and protein binding was not statistically significant between groups. Assuming an MIC of 1 mg/L for MRSA, *f*T_>1xMIC_ was 99.6% ± 0.65% in the intermittent group and 100% ± 0% in the continuous group.

### 3.4 Subcutaneous parasternal pharmacokinetics

Subcutaneous microdialysis was feasible in all patients with a mean probe recovery of 11.8% ± 6.5% (mean ± SD). In the intermittent group, ceftaroline peak subcutaneous concentrations during steady state were 2.6 ± 1.5 mg/L (left parasternal) and 5.3 ± 2.3 mg/L (right parasternal). In the continuous group, average steady state subcutaneous concentrations were 2.7 ± 1.4 (left parasternal) and 2.5 ± 1.6 mg/L (right parasternal), respectively. In the intermittent group, AUC_SS 0–24_ was 38.2 ± 20 (mg x h)/L (left parasternal) and 65.5 ± 13.1 (mg x h)/L (right parasternal), respectively. In the continuous group AUC_SS 0–24_ was 48.5 ± 34.8 (left parasternal) and 36.4 ± 27.5 (mg x h)/L (right parasternal), respectively. The difference in AUC_SS 0–8_ and AUC_SS 0–24_ was not statistically significant between groups.

Assuming an MIC of 1 mg/L for MRSA, *f*T_>MIC_ was 93% ± 9.6% (left parasternal) and 98.7% ± 2.2% (right parasternal) in the intermittent group. In the continuous group, *f*T_>MIC_ was 75% ± 50% (left parasternal) and 86% ± 26.6% (right parasternal).

### 3.5 Target attainment

**FIGURE 3:**
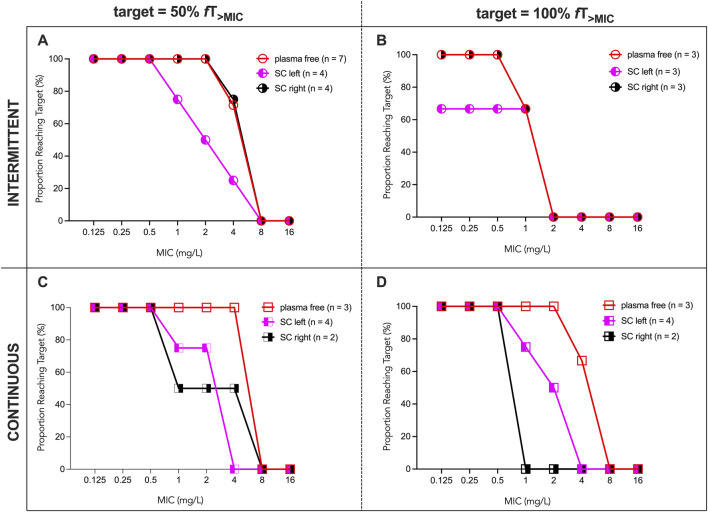
Proportion of participants reaching the target 50% *f*T_>MIC_ and 100% *f*T_>MIC_. The proportion of patients reaching the target is shown for MICs 0.125–16 mg/L. The number of patients included in each analysis after application of the predefined exclusion criteria are indicated in the legend. **(A)** PRT for free ceftaroline in plasma (open red circles) and free ceftaroline in the right (black half-filled circles) and left (magenta half-filled circles) parasternal SC tissue in the intermittent group for the target 50% *f*T_>MIC_. **(B)** PRT for free ceftaroline in plasma (open red circles) and free ceftaroline in the right (black half-filled circles) and left (magenta half-filled circles) parasternal SC tissue in the intermittent group for the target 100% *f*T_>MIC_. **(C)** PRT for free ceftaroline in plasma (open red boxes) and free ceftaroline in the right (black half-filled boxes) and left (magenta half-filled boxes) parasternal SC tissue in the continuous group for the target 50% *f*T_>MIC_. **(D)** PRT for free ceftaroline in plasma (open red boxes) and free ceftaroline in the right (black half-filled boxes) and left (magenta half-filled boxes) parasternal SC tissue in the continuous group for the target 100% *f*T_>MIC_. MIC = minimum inhibitory concentration; PRT, proportion of patients reaching the PK/PD target; SC, subcutaneous.

We evaluated the proportion of participants reaching the PK/PD targets of 50% *f*T_>MIC_ and 100% *f*T_>MIC_ (Figure 3) at increasing MICs. The number of participants included in this analysis after application of the predefined exclusion criteria for missing concentration-time data are indicated in the legends of Figure 3.

Considering free plasma concentrations, all participants reached the target 50% *f*T_>MIC_ for MICs up to 2 mg/HL in the intermittent group, whereas all participants in the continuous group achieved this target at MICs up to 4 mg/L. More than 60% of patients achieved 100% *f*T_>MIC_ for MICs up to 1 mg/L in the intermittent group, whereas all patients in the continuous group achieved this target for MICs up to 2 mg/L.

In subcutaneous tissues the proportion of participants achieving 50% *f*T_>MIC_ was ≥75% at MICs up to 1 mg/L in both groups, except at the right parasternal subcutaneous site under continuous infusion, where 50% reached the target. At MICs above 1 mg/L, target attainment decreased markedly, often reaching zero at MICs ≥2 mg/L. The proportion of patients achieving 100% *f*T_>MIC_ was lower overall, with substantial declines starting at MICs of 1 mg/L.

## 4 Discussion

This prospective, randomized pharmacokinetic study evaluated intra- and postoperative plasma and parasternal subcutaneous ceftaroline concentrations in patients undergoing cardiac surgery with CPB receiving either intermittent or continuous administration. To our knowledge, this is the first study to measure subcutaneous ceftaroline concentrations using microdialysis in this perioperative setting.

For ceftaroline, % *f*T_>MIC_ correlates with antimicrobial efficacy (Andes and Craig, 2006; Lodise et al., 2006). Early *in vivo* data established that 26% *f*T_>MIC_ is sufficient for stasis, 33% for 1 log10 kill, and 45% for 2 log10 kill (Andes and Craig, 2006). Subsequent *in vitro* dilutional and hollow-fiber models confirmed these thresholds across a broader range of *S. aureus* isolates with MICs up to 4 mg/L, including SCCmec types and PBP2a variants (MacGowan et al., 2013; Singh et al., 2017). Accordingly, 50% *f*_T>MIC_ may be considered a reasonable plasma PK/PD target, while higher targets may still be relevant when tissue penetration is impaired or high-MIC pathogens are encountered (Das et al., 2019).

Plasma concentrations consistently exceeded the pharmacodynamic threshold for MRSA at an MIC of 1 mg/L (Das et al., 2019; The European Committee on Antimicrobial Susceptibility Testing, 2025). Consistentwith the time-dependent PK/PD properties of beta-lactam antibiotics, in which efficacy is driven by the duration of free drug concentrations above the MIC, continuous infusion was associated with higher plasma target attainment at higher MICs compared with intermittent infusion.

Subcutaneous concentrations were considerably lower and demonstrated high interindividual variability. At an MIC of 1 mg/L, subcutaneous % *f*T_>MIC_ was generally high under intermittent administration but showed substantially greater variability under continuous infusion. At higher MICs, tissue exposure frequently did not reach plasma-based PK/PD targets irrespective of the mode of administration. This suggests that tissue penetration may represent the limiting factor for antimicrobial exposure at the surgical site in this perioperative setting. Interestingly, subcutaneous concentrations were descriptively higher in the intermittent infusion group, raising the hypothesis that the efficacy of continuous infusions may also depend on the target tissue and that transient plasma peaks may facilitate tissue penetration. Similarly, continuous infusion resulted in lower cerebrospinal fluid concentrations compared with intermittent infusion in the NeoMero studies (Germovsek et al., 2018). These observations may help explain why randomized controlled trials in septic patients and patients with pneumonia do not always demonstrate better clinical outcomes with continuous beta-lactam infusions (Dulhunty et al., 2015; Dulhunty et al., 2024; Lee et al., 2018; Monti et al., 2023).

In contrast to published data (Andreas et al., 2013), we found no significant difference between left and right parasternal s. c. concentrations (intermittent group, *P = 0.81*; continuous group, *P = 0.36*), despite left internal mammary artery harvesting during all procedures. This finding may be related to differences in surgical technique, including the use of different sternal retractors with more homogenous compression of parasternal tissues.

Taken together, our findings demonstrate that attainment of established plasma-based PK/PD targets may overestimate drug exposure at the surgical site, particularly under conditions of altered perioperative PK. However, there are no validated, evidence-based PK/PD targets for subcutaneous tissues. The clinical implications of this observation are uncertain and require clinical studies correlating tissue exposure with clinical outcomes.

Matzneller et al. studied ceftaroline PK in plasma and subcutaneous tissue in healthy volunteers (Matzneller et al., 2016). The study demonstrated that subcutaneous concentrations remained below plasma levels, with subcutaneous tissue-to-plasma ratios of 0.75 ± 0.3 after repeated dosing. While plasma PK was comparable to healthy volunteers (Riccobene et al., 2013; Justo et al., 2015; Matzneller et al., 2016), cardiac surgery patients in this study had lower tissue-to-plasma ratios, ranging from 0.4-0.7 with significant interindividual variability. Similarly, we observed considerable inter- and intraindividual variability in parasternal subcutaneous tissue concentrations. This discrepancy likely reflects the altered microvascular perfusion during cardiac surgery with CPB, vasoactive drug administration, and mechanical effects exerted by sternal retractors together with the patients’ cardiovascular comorbidities. Our findings demonstrate the limitations of extrapolating PK data from healthy volunteers and highlight the importance of measuring tissue concentrations when evaluating dosing strategies in the perioperative setting.

Furthermore, PK factors such as protein binding may contribute to variability in tissue exposure. Ceftaroline exhibits low and concentration-dependent protein binding ranging from approximately 14%–28% (Jorgenson et al., 2011). Higher peak concentrations may increase the free drug fraction and enhance tissue diffusion. In the present study, protein binding was measured in each patient to derive free plasma concentrations. However, serial measurements over the entire study period were not performed. Although physiologic alterations during cardiac surgery, such as hemodilution and changes in albumin concentration, could theoretically influence protein binding, the overall variability of protein binding is relatively limited compared with highly protein bound agents such as cefazolin (Vella-Brincat et al., 2007). Importantly, tissue concentrations were quantified using *in vivo* microdialysis, which directly measures free drug concentrations and therefore accounts for any concentration-dependent changes in protein binding.

Current guidelines recommend the combination of vancomycin plus a β-lactam as surgical prophylaxis for MRSA-colonized patients undergoing cardiac surgery (Jeppsson et al., 2024). Vancomycin must be administered 2 h before incision and is associated with nephrotoxicity and the potential for poor tissue penetration (Martin et al., 1994; Skhirtladze et al., 2006). Ceftaroline provides antimicrobial activity against MRSA, can be administered closer to incision (30–60 min) and demonstrates favorable plasma PK. While the present study was not designed to assess clinical efficacy, our findings support further investigation of the currently approved high-dose regimen in patients with risk factors for MRSA infections.

Some limitations must be acknowledged. First, no direct correlation with surgical site infection rates or other clinical outcomes was assessed, which precludes assessment of clinical efficacy. Second, the sample size of seven patients per group and the variability of patient demographics may limit the generalizability of our results. Third, we reported proportions achieving PK/PD targets based on observed data rather than conducting formal probability of target attainment analyses, which would better account for variability in PK, dosing regimens, and MIC distributions.

In conclusion, this study provides the first data on subcutaneous ceftaroline exposure in patients undergoing cardiac surgery with CPB. Plasma target attainment was consistently achieved, whereas subcutaneous exposure was highly variable and not uniformly improved by continuous infusion. These findings underscore the importance of measuring tissue concentrations when evaluating antibiotic exposure in the perioperative setting and highlight that improved plasma exposure does not always translate into superior tissue exposure. Further clinical studies integrating tissue exposure with clinical efficacy are needed to define optimal dosing strategies in this setting.

## Data Availability

The raw data supporting the conclusions of this article will be made available by the authors upon reasonable request, in accordance with institutional and data protection regulation.
